# Detection of *Babesia odocoilei* in Humans with Babesiosis Symptoms

**DOI:** 10.3390/diagnostics11060947

**Published:** 2021-05-25

**Authors:** John D. Scott, Muhammad S. Sajid, Emily L. Pascoe, Janet E. Foley

**Affiliations:** 1 Department of Medicine and Epidemiology, School of Veterinary Medicine, University of California Davis, Davis, CA 95616, USA; drsohailuaf@uaf.edu.com (M.S.S.); elpascoe@ucdavis.edu (E.L.P.); jefoley@ucdavis.edu (J.E.F.); 2 Faculty of Veterinary Medicine, University of Agriculture, Faisalabad 38040, Pakistan

**Keywords:** *Babesia odocoilei*, piroplasm, human babesiosis, 18S rRNA, ticks, *Ixodes scapularis*, parasite, white-tailed deer, molecular identification, human pathogen

## Abstract

Human babesiosis is a life-threatening infectious disease that causes societal and economic impact worldwide. Several species of *Babesia* cause babesiosis in terrestrial vertebrates, including humans. A one-day clinic was held in Ontario, Canada, to see if a red blood cell parasite, which is present in blacklegged ticks, *Ixodes scapularis*, is present in humans. Based on PCR testing and DNA sequencing of the 18S rRNA gene, we unveiled *B. odocoilei* in two of 19 participants. DNA amplicons from these two patients are almost identical matches with the type strains of *B. odocoilei* in GenBank. In addition, the same two human subjects had the hallmark symptoms of human babesiosis, including night sweats, chills, fevers, and profound fatigue. Based on symptoms and molecular identification, we provide substantive evidence that *B. odocoilei* is pathogenic to humans. Dataset reveals that *B. odocoilei* serologically cross-reacts with *Babesia duncani*. Clinicians must realize that there are more than two *Babesia* spp. in North America that cause human babesiosis. This discovery signifies the first report of *B. odocoilei* causing human babesiosis.

## 1. Introduction

Human babesiosis (human piroplasmosis) is potentially a life-threatening zoonotic infection. This infectious disease is caused by a red blood cell parasite belonging to the genus *Babesia* (Apicomplexa: Piroplasmida: Babesiidae). Historically, this unicellular intraerythrocytic piroplasm was first discovered in 1888 by Victor Babes, a Romanian pathologist [1] and, five years later, Smith and Kilborne found that ixodid ticks (Acari: Ixodidae) are the actual vectors which transmit *Babesia* spp. to terrestrial vertebrate hosts [2]. Inevitably, the first case of human babesiosis was reported in a splenectomized Croatian cattle farmer in 1956 [3], and later named *Babesia divergens*.

In North America, human babesiosis was first recognized in a splenectomized Californian man in 1966 [4], and this intracellular hemoparasite was subsequently named *Babesia duncani*. One year later, *Babesia microti* was described in a middle-aged, non-splenectomized woman residing on Nantucket Island, Massachusetts [5]. *Babesia* spp. are typically transmitted by ticks but may also be transmitted by blood transfusion [6,7], organ transplantation [8], and maternal-fetal transmission [9,10,11,12]. Human babesiosis infection can range from asymptomatic to mild, malaria-like symptoms to a disabling, insidious disease that may result in death. Clinical manifestations of babesiosis often include night sweats, fevers, flushing, chills, heat and cold intolerance, profound fatigue, muscle aches, air hunger, increased thirst, headaches, sleep disturbance, and anxiety [13,14]. Moreover, hemolysis, thrombocytopenia, and dysautonomia are common pathology. Patients that are asplenic or infected with another tick-borne zoonotic pathogen can be more symptomatic with severe pathological sequelae [15]. Around the globe, human babesiosis is caused by known *Babesia* spp. that include, but are not limited to, *B. crassa, B. divergens*, *B. duncani* (WA1)*, B. microti, B. venatorum,* *Babesia* sp. variants CA1, CA3, and CA4, *Babesia* sp. CN1, *Babesia divergens*-like MO1, and *Babesia* sp. TW1 [3,4,5,16,17,18,19,20].

The blacklegged tick, *Ixodes scapularis*, is the prime vector of *B. odocoilei* [21]. This tick species has transstadial passage (larva to nymph or nymph to adult) and transovarial transmission (female to eggs to larvae) of *B. odocoilei* [22,23]. Since the midgut is the only tick organ that is retained during the molt [24], larvae and nymphs can maintain *B. odocoilei* gametes/ray bodies throughout the molt. After the molt, kinetes can be transferred to the tick salivary glands and the ovaries. In particular, *I. scapularis* females can transmit kinetes to their eggs and, upon hatching, forward to the larvae. During engorgement, *Babesia*-infected *I. scapularis* larvae, nymphs, and females can transmit infective sporozoites to their hosts, including humans. Consequentially, blacklegged ticks can perpetuate *B. odocoilei* in an ongoing enzootic transmission life cycle.

Perry et al. provide the first report of *B. odocoilei* in a white-tailed deer, *Odocoileus virginianus*, which is a reservoir host [25]. Initially, *B. odocoilei* was generally considered to be non-pathogenic to white-tailed deer [26]; however, this obligate, intracellular parasite was found pathogenic to stressed and compromised cervid hosts [27], and some have fatal outcomes. Ecologically, white-tailed deer typically support the reproduction and propagation of blacklegged ticks. From an epizootic standpoint, *B. odocoilei* can cause cervine babesiosis in white-tailed deer and other cervids [27,28,29]. This piroplasm has also been detected in bovids (i.e., desert bighorned sheep, *Ovis canadensis nelsoni*) which are indigenous in far-western North America [30,31]. Since *B. odocoilei* has been detected in the western blacklegged tick, *Ixodes pacificus*, this ixodid tick may be a vector infecting bovids, and possibly other mammals, including humans [32].

The distribution of *B. odocoilei* in North America is continent-wide and reported as far south as Texas [25,27] and as far north as northern Saskatchewan [28]. East of the Rocky Mountains, *I. scapularis* has been documented as far north and west as northwestern Alberta and as far north and east as the southern part of Newfoundland and Labrador [33]. Biogeographically, *B. odocoilei* was detected in *I. scapularis* larvae and nymphs collected from songbirds in Ontario and Quebec, and thus, this apicomplexan piroplasm has widespread geographical distribution [33,34,35,36,37,38]. Questing *B. odocoilei*-positive *I. scapularis* adults (males, females) have also been collected by flagging low-lying vegetation in Ontario [34,35,36].

Wildlife habitats where white-tailed deer (the reservoir) and the blacklegged tick (the vector) co-exist are focal areas for the enzootic transmission cycle of *B. odocoilei* [37]. Additionally, songbirds widely disperse *B. odocoilei*-infected *I. scapularis* larvae and nymphs across the North American landscape, especially during spring and fall migrations [33,34,36,37]. During temperate weather (above 0 °C and free of snow cover), *B. odocoilei*-infected *I. scapularis* ticks may be host-seeking in wooded areas, including parks [38].

The principal aim of this human *Babesia* study was (1) to establish whether the microscopic parasite *B. odocoilei* is present in humans and (2) to ascertain if this apicomplexan piroplasm is pathogenic to humans.

## 2. Results

In total, 19 subjects participated in this human *Babesia* study. The age ranged from 16 to 76 years and consisted of 6 males and 13 females. Both Subject 1 and Subject 2 had all seven symptoms listed on the questionnaire and the consent form.

In all, *B. odocoilei* DNA was detected in the two subjects. The DNA amplicons and their associated GenBank accession numbers are listed in Table 1.

Subject 1: A 23-year-old female did not recall a tick bite and was previously diagnosed clinically with human babesiosis. A standard regimen of atovaquone (Mepron) was administered. After temporary relief from treatment, she continued to encounter common symptoms associated with human babesiosis, namely, night sweats, chills, fevers, profound fatigue, increased thirst, muscle aches, and sleep disturbance. She had her spleen intact (non-splenectomized). When she was tested for *Babesia* spp., using molecular characterization, she was positive for *B. odocoilei* (MW368483).

Subject 2: A 74-year-old male was bitten by an *I. scapularis* nymph prior to onset of symptoms. He developed familiar *Babesia* symptoms, namely, night sweats, chills, fevers, profound fatigue, increased thirst, muscle aches, and sleep disturbance. He had serology testing done at a Clinical Laboratory Improvement Amendments (C.L.I.A.)-accredited laboratory and was serologically positive for *B. duncani* and, also, positive by fluorescence *in situ* hybridization (FISH) for *Babesia*. He was treated with a combination of atovaquone (Mepron) and azithromycin, and clinical symptoms abated temporarily and then relapsed. Next, he was prescribed atovaquone and proguanil (Malarone), and again, his symptoms subsided provisionally but returned. He also employed a derivative of *Artemisia annua*, an herbal medication used for human babesiosis, but it failed to resolve the symptoms. This subject had his spleen intact. When he was tested for *Babesia* spp. using molecular analysis, he was positive for *B. odocoilei* (MW368482).

DNA amplicon sequences of Subject 1 and Subject 2 had one and two mismatches in 428 base pairs (bp) and 440 bp, respectively (Table 1). These human-derived DNA amplicons very closely matched *B. odocoilei* type strains in GenBank (Table 1). The similarity to the type strains of *B. odocoilei* was 99.55% and 99.77%.

**Figure 1:**
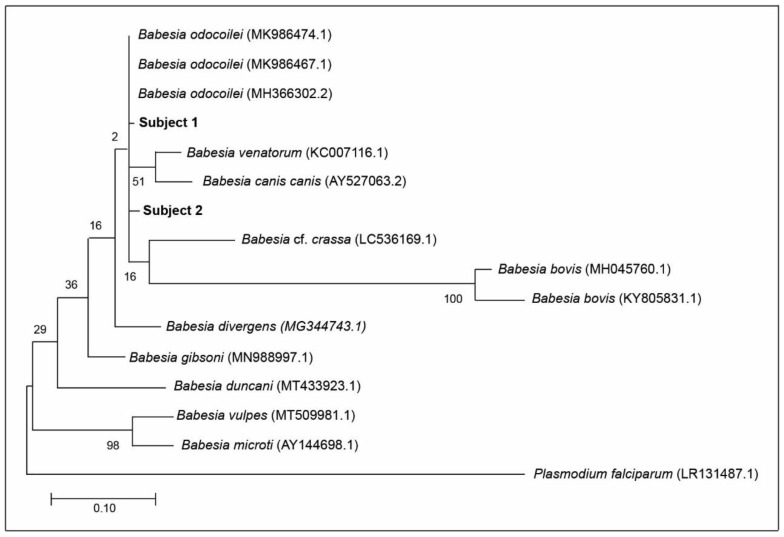
Maximum likelihood phylogenetic tree of 18S rRNA sequences amplified from human blood with 13 published sequences of ten different reference *Babesia* species for comparison, plus *Plasmodium falciparum* as an outgroup, represent the relationship of Subject 1 and Subject 2 to other *Babesia* spp. Alphanumeric values in brackets denote published GenBank sequences. The scale bar represents the percentage of genetic variation along the tree branches.

Based on DNA sequencing and Basic Local Alignment Search Tool (BLAST) analysis, which is available online at https://blast.ncbi.nlm.nih.gov (accessed on 20 December 2020), all of the amplicons obtained from the pair of apicomplexan-specific primers were negative for *B. duncani*. Furthermore, based on 100 BLAST rounds, there was no trace of molecular evidence for *B. duncani* in the blood sample of Subject 2. Using the phylogenetic tree, the distance between *B. odocoilei* (for Subject 2) and *B. duncani* is 90.67% (Figure 1).

An E-value of 0.0 for Subject 1 and Subject 2 demonstrates that the very close matches (i.e., 99.77% and 99.55%, respectively) to *B. odocoilei* is highly significant.

## 3. Discussion

*Babesia odocoilei* is one of several *Babesia* species worldwide that cause human babesiosis. In the present study, we provide confirmatory molecular evidence of human babesiosis caused by *B. odocoilei*. None of the human subjects in this study had lived in or knowingly visited an area endemic for *B. odocoilei*. Molecular identification was used to confirm the presence of *B. odocoilei* in subjects exhibiting the classical symptoms of human babesiosis. We provide the first ever report of *B. odocoilei* infecting humans.

### 3.1. Meeting Ethical Requirements for Babesia Study

This human *Babesia* study directly involved human subjects rather than using routine animal modeling. This methodology has eliminated animal testing, sparing hundreds of vertebrates unnecessary suffering and death. Animal testing, which would utilize a group of mammals, such as rhesus macaques, is not required. In the present study, there were no lab animals involved other than humans who were willing participants. Importantly, this study is unique because it directly samples and tests human erythocytes where *Babesia* reside. At the same time, collection of blood draw samples by routine venipuncture was minimally invasive. Testing of laboratory animals would be invasive, labor intensive, and expensive for this type of biomedical research. In addition monitoring and determining the immune responses of all subjects would be a complex and in-depth process and was not part of this study. Moreover, it would be unethical to solicit humans and inoculate them with *B. odocoilei*, which is an intraerythrocytic parasite and then perform a timeline (i.e., implement pathogen inoculation, conduct antimicrobial challenge, assess clinical results). Such a study would violate the Declaration of Helsinki, the Nuremberg Code, and the Belmont Report. The manner in which we have conducted this study meets the basic tenets of ethical biomedical studies. We received approval from the Western Institutional Review Board (WIRB) based on the fact that our protocol is highly ethical and meets the requirements of a human study. In line with standard biomedical protocol (i.e., honesty, transparency, integrity), we had an ethical obligation to notify subjects privately who were positive for *Babesia*. Regarding the E-value, which was 0.0 for each subject, we are highly confident about the presence of *B. odocoilei* DNA in the blood samples. Not only does our study meet the professional policy of WIRB, but our methodology meets the epitome of an ethical human *Babesia* study.

### 3.2. Age as a Factor

Subject 1 was aged 23, and her night sweats would not have been caused by menopause. The average age of a North American menopausal woman is 51 years. Although Subject 2 was above middle age, his immune response would depend on the vivacity of his immune system and the level of babesial parasitemia. Regardless of age, both subjects were infected with an apicomplexan-confirmed parasite, *B. odocoilei*, and both exhibited classic symptoms of human babesiosis.

### 3.3. Source of B. odocoilei Infection

Since Subject 1 did not remember a tick bite, the putative mode of actual transmission of *B. odocoilei* is unclear. Only 14–41% of patients have recollection of being bitten by a tick [39,40]. Subject 2 was bitten by an *I. scapularis* nymph that was identified by an acarologist specializing in ticks. Although the fully engorged nymph was not tested for *B. odocoilei*, this nymphal tick would most likely have been the source of *B. odocoilei* infection. All the mobile developmental life stages (larvae, nymphs, adults (females)) of blacklegged ticks are competent vectors of *B. odocoilei* [21,37,38]. Moreover, transovarial transmission of *B. odocoilei* normally occurs when an infected, gravid female lays her eggs that hatch to larvae. The ecology and epidemiology of *B. odocoilei* in North America is much more widespread in the natural environment than previously conceptualized.

### 3.4. Testing as a Factor

Symptoms typically provide the basis for a clinical diagnosis; however, patients can be asymptomatic and still have a babesial infection. In this study, Subject 1 and Subject 2 had all of the seven symptoms listed on the questionnaire and consent form. Prior to blood sampling day (BSD) at the one-day clinic, Subject 1 had a clinical diagnosis of babesial infection and was treated with standard antimicrobials accordingly (Table 1). Although standard testing was not performed, clinical expertise is an integral part of evidence-based medicine. With respect to Subject 2, his initial diagnosis was based on positive serology for *B. duncani* from a licensed clinical laboratory. This male also had all of the clinical manifestations listed on the subject questionnaire and consent form and was administered antimicrobials (Table 1). In addition, Subject 2 was positive for the fluorescent *in situ* hybridization (FISH) assay, which is a genus-specific test for *Babesia* [41]. FISH is not species-specific and simply represents *Babesia*. Collectively, Subject 2 expresses four pathways (i.e., pre-BSD serology, post-BDS serology, FISH, molecular identification) and validates *Babesia* positivity for human babesiosis.

Both Subject 1 and Subject 2 had symptoms consistent with active babesial infection. Pathologically, both subjects had night sweats which are the trademark of human babesiosis. Because malaria and human babesiosis have similar symptoms (e.g., night sweats, chills, profound fatigue), patients are often thought to have malaria. With these malaria-like symptoms, clinicians may hastily think that the patient has an infection of *Plasmodium falciparum* (Phylum: Apicomplexa), which is the etiological microorganism of malaria. If *P. falciparum* would have been the cause of night sweats in Subject 1 and Subject 2, the pair of apicomplexan-specific primers would have detected this apicomplexan parasite. No such microorganism was detected in either subject. Instead, an apicomplexan babesial piroplasm, *B. odocoilei*, was identified in both subjects. Researchers indicate that *Babesia* spp. cannot be recognized by size [42,43]. Therefore, trying to identify *Babesia* spp. morphologically is difficult. Because of the uncertainty of using microscopy, morphology, and immune response testing, we capitalized on using molecular identification, namely, a pair of aplicomplexan-specific primers, followed by DNA sequencing and BLAST analysis to confirm the babesial piroplasm, *B. odocoilei*. When BLAST analysis was conducted, neither Subject 1 nor Subject 2 had DNA amplicons for *B. duncani* (Table 1).

### 3.5. Serology as a Factor

Subject 1, who was clinically diagnosed with human babesiosis, did not have any laboratory serology testing for *Babesia*. In the case of Subject 2, he was serologically positive for *B. duncani* prior to BSD and seroreactive post-BSD. Based on meta-analysis of other studies, serology for *Babesia* is unreliable and lacks assurance of differentiation [7,14,44,45,46]. This shortfall is compounded by the fact that there are at least 111 valid species of *Babesia* worldwide [43]. Since there is a wide diversity of *Babesia* species globally, authentication hinges on molecular identification. In North America, there currently is only serology testing for two *Babesia* species, namely, *B. duncani* and *B. microti.* In the case of Subject 2, two-choice serology failed to properly delineate the actual identification of the causal organism. The present human *Babesia* study shows that current serological testing has critical limitations that may lead to spurious results and, ultimately, unresolved treatment regimens (Table 1).

### 3.6. Cross-Reactivity

Subject 2 was serologically positive for *B. duncani* 36 mo prior to BSD and was seroreactive for *B. duncani* 6 mo after BSD. With the combination of BSD nucleic acid detection and pre- and post-BSD serological testing, we were assuredly able to discern that *B. odocoilei* cross-reacts with *B. duncani*. These serological findings demonstrate that patients with *B. odocoilei* can be mislabeled with having *B. duncani*. When patients are serologically positive for *B. duncani* and, in fact, are positive for *B. odocoilei*, such results are not only misleading but misrepresent the causal organism. On a wider scale, piroplasm researchers have found that serological testing is not only capricious but spurious [44,45,46]. Misdiagnosis is inevitable. Our 18S rRNA testing and BLAST analysis verifies that *B. odocoilei* can cross-react with *B. duncani*. If *B. duncani* was present in Subject 2, it would have been picked up by the pair of apicomplexan-specific primers and appeared during the BLAST analysis. Specifically, *B. duncani* was not detected in 100 BLAST replications. Moreover, *B. duncani* is considerable distance phylogenetically from *B. odocoilei* and not in the same genetic clade (Figure 1).

### 3.7. Blood Smear Performance

None of the *Babesia*-positive subjects reported a blood smear on the questionnaire. Although blood smears are considered by some to be the gold standard for *Babesia* testing, the pitfalls of blood smears are well documented [18,44,46,47]. *Babesia* parasites are too small to be definitively diagnosed by this method [42,45]. Researchers have found that approximately 1% of red blood cells are normally infected with *Babesia* and indicate that blood smears are highly inaccurate and miss up to 95% of positive cases [47]. Even the best lab technicians have a hard time picking up miniscule *Babesia* parasites [14,18,44,45,46,47]. Not only are blood smears very inefficient, but they are very time consuming, especially when individuals have low parasitemia.

### 3.8. Virulence of B. odocoilei

Elevated *Babesia* parasitemia in humans can result in fatal outcomes [3,5,19]. Clinicians indicate that *B. duncani* is more virulent than *B. microti*, and more difficult to treat [48,49,50]. Derived from the analytical revelations from our study, *B. duncani* could well be *B. odocoilei* in some cases. Anchored in our molecular findings, we suggest that clinicians may, in reality, be dealing with *B. odocoilei* rather *B. duncani* or other *Babesia* spp. During routine testing, *B. duncani* may actually be *B. odocoilei* in disguise. Subject 2 experienced what was thought to be *B. duncani* but was actually *B. odocoilei*. Although not the only prominent sign and symptom, the hallmark sign of human babesiosis is recurrent drenching night sweats [14,47].

### 3.9. Contracting Human Babesiosis in Non-Endemic Areas

Some medical professionals contend that people must frequent an endemic area to contract human babesiosis [18]. However, tick researchers have shown that visiting an endemic area is not required to become infected with *B. odocoilei* [37,38]. This epidemiological factor is exacerbated by the fact that north-bound, migratory songbirds widely disperse *B. odocoilei*-infected *I. scapularis* larvae and nymphs [33,35,36,38]. Even though there are no *B. odocoilei* risk maps, such maps could be very misleading. Furthermore, other modes of transmission occur. Neither Subject 1 nor Subject 2 lived in a known endemic area for *B. odocoilei*. In North America, there are at least nine different tick-borne pathogens/pathogen groups, including *Babesia* species [23,37,38].

### 3.10. Persistence of B. odocoilei

Both Subject 1 and Subject 2 continued to be symptomatic for human babesiosis after standard antimicrobial treatment. Using a licensed clinical laboratory, Subject 2 tested serologically positive for *B. duncani* and also positive for *Babesia* by FISH, a genus-specific *Babesia* test [41]. Both of these tests were conducted 36 mo prior to BSD. Standard antimicrobial treatments were administered during this 36 mo period. Although *Babesia* symptoms subsided with traditional therapy, clinical manifestations of human babesiosis returned within 4 d after the patient had finished the full dosage. Subject 2 was seroreactive for *B. duncani* and, despite further *Babesia* treatment, continued to be symptomatic. Subject 2 was unsuccessful in obtaining a cure with standard regimens, namely, atovaquone plus azithromycin and, likewise, atovaquone coupled with proguanil. Based on the current availability of antimicrobials, this recrudescent pattern of symptoms after treatment modalities indicates that certain babesial infections can be persistent in the human body [14,19,49,50,51]. Founded on the ongoing clinical sequelae of Subject 1 and Subject 2, *B. odocoilei* can induce a recalcitrant infection and has a strong predilection to be persistent. Persistence of *B. odocoilei* lasted for more than 3.5 years in Subject 2 despite treatment modalities with standard anti-*Babesia* therapy. As experienced by other human babesiosis patients, Subject 2 experienced ongoing treatment failure [49,50,52,53]. Subject 1 and Subject 2 both had persistent and recrudescent babesiosis, which is often associated with this infectious disease [49,50,51,52,53]. Other *Babesia* spp. have been reported to exhibit resistance to traditional treatment suggesting the necessity of developing new antimicrobials [52,53,54,55,56]. As evident in the present study, anti-*Babesia* regimens may result in treatment failure of human babesiosis patients [14,48,49,50,51,52,53,54,55,56].

### 3.11. Molecular Identification

DNA sequencing and BLAST analysis provide a new molecular means of detecting *Babesia* species and offered a precise mode for detecting *B. odocoilei* in human blood. A pair of apicomplexan-specific primers followed by DNA sequencing and BLAST analysis provided confirmation of *B. odocoilei* in Subject 1 and Subject 2. Although Subject 1 and Subject 2 had one and two mismatches (Figure 1), respectively, they had very close proximity to the type strains in GenBank (Table 1). Due to mutations in nature over time, mismatches from the archetypical strains are ordinary. Since standard testing methods (e.g., serology, blood smears, FISH) convey unreliable results [14,18,44,45,46,47], molecular characterization is indispensable in distinguishing between *Babesia* species [42]. In essence, molecular identification has been the linchpin in solving the longstanding question of whether *B. odocoilei* is pathogenic to humans.

### 3.12. Number of Subjects

Although we had quite a limited number of participants, we were still able to confirm *B. odocoilei* in human subjects. Previously, some early cases of human babesiosis were based on a single human case, such as *B. divergens* [3], *B. duncani* [4], *B. microti* [5], and *Babesia* sp. MO1 [19]. In some of these seminal cases, *Babesia* spp. were acknowledged and affirmed because the patients died. In the case of the first three *Babesia* species listed, PCR and DNA sequencing were not discovered; however, the differences in these *Babesia* species were recognized. Based on phylogeny [38], *B. odocoilei* is in the same sister group as those of *B. divergens* and *B. venatorum* which are both pathogenic to humans (Figure 1) [44,45]. Our flagship discoveries elucidate the fact that we provide the first human *Babesia* study using molecular methodology and we also present key data that *B. odocoilei* can serologically cross-react other *Babesia* species and be pathogenic to humans.

## 4. Materials and Methods

### 4.1. Participation in Study

An open invitation to attend a one-day *Babesia* clinic was extended to the public via networking, and participants came of their own free will. On arrival at the healthcare center, they were informed about the human *Babesia* study, and asked to read the instructions. If they wished to participate, they filled out the questionnaire and signed the consent form. Diagnosis, treatment, and dosage are outside the domain of this human *Babesia* study.

### 4.2. Collecting Blood Samples

A professional phlebotomist drew two tubes (4.5 mL each) of blood via customary venipuncture from each of the participants. These tubes contained ethylenediamine tetraacetic acid (EDTA) to keep blood from clotting. After blood draw, subjects were free to continue with their normal daily activities and, henceforth, with their medical care with his/her healthcare provider. Blood samples were kept on a freezer pack in a cooler until they could be sent to the lab, directly by overnight courier scheduled to arrive the next morning. In order to maintain confidentiality, all of the blood samples had a two-step, de-identification process (first by a 3-letter code, and later by a number code). Participant personal history was used only for purposes described in the consent form. Even though not required, some participants had prior testing for human babesiosis at a licensed clinical laboratory specializing in testing for tick-borne zoonotic diseases.

### 4.3. Informed Consent

Each willing subject completed a human *Babesia* study questionnaire providing background information and voluntary consent. Each subject signed the consent form to ensure that his/her personal information would be kept confidential. The signed informed consent forms to collect blood and publish the results were obtained in accordance with the ethics approval requirements of the Western Institutional Review Board (W.I.R.B.), Puyallup, WA, U.S.A. (Study Number 1294045). Using number coding, WIRB specified that the results were to be published in a peer-reviewed scientific article. The present study follows basic tenets laid out in the Declaration of Helsinki, the Nuremberg Code, the Belmont Report, and the Collaborative Institutional Training Initiative (C.I.T.I.) program. Responsible conduct of biomedical ethical research includes integrity, honesty, and transparency.

### 4.4. Babesia Detection

DNA was extracted from human blood using the Qiagen DNeasy Blood and Tissue Kit (Qiagen, Valencia, CA, USA) following the manufacturer’s protocol for blood. DNA was stored at −20 °C until PCR was performed. The BJ1 (5′-GTC-TTG-TAA-TTG-GAA-TGA-TGG-3′) and BN2 (5′-TAG-TTT-ATG-GTT-AGG-ACT-ACG-3′) primers were used to amplify the 18S rRNA gene of *Babesia* using PCR conditions previously described [34,45]. Protocol modifications included a greater volume of erythrocyte DNA (~5 ng/µL). Specifically, 5 µL of DNA was used instead of 2.5 µL, whereas 2.5 µL of H_2_O was applied instead of 5 µL. In total, not more than 25 µL was utilized for the PCR reaction mixture. The resulting amplicons were visualized on 1% agarose gel containing GelStar nucleic acid stain (Lonza, Rockland, ME, USA), and the *Babesia* amplicons that were 420–490 bp in length were excised from the gel and prepared for DNA sequencing using the QIAamp DNA Kit (Qiagen, Valencia, CA, USA). DNA sequencing was performed at the University of California Davis Sequencing Facility using the Big Dye Terminator cycle sequencing kit (Applied Biosystems, Foster City, CA, USA) and BJ1 and BN2 primers.

Sequences were compared to those published in GenBank using the BLAST database search program (https://blast.ncbi.nlm.nih.gov/Blast.cgi#blank (accessed on 20 December 2020)). In addition, sequences were manually corrected for ambiguous base calls and end-reading errors. All DNA sequences were trimmed to approximately the same length, and the MUSCLE algorithm performed the sequence alignments [57]. DNA sequences from the *Babesia* species (*B. odocoilei*, *B. bovis*, *B*. cf. *crassa*, *B. divergens*, *B. duncani*, *B. canis canis*, *B. gibsoni*, *B. microti*, *B. vulpes*, and *B. venatorum*) were from GenBank for inclusion in the phylogenetic tree. *Plasmodium falciparum* was used as an outgroup. Phylogeny was resolved using the maximum likelihood method in MEGA 10.0.5 [58] and, similarly, using the General-Time-Reversible model with gamma distribution as determined by jModeltest 2.1.10 [59]. Bootstrapping was performed based on 1000 pseudoreplicate datasets generated from the original sequence alignments.

## 5. Conclusions

We show that *B. odocoilei*, a tick-borne zoonotic species, is pathogenic to humans. Since *B. duncani* cross-reacts with *B. odocoilei*, North American patients who test serologically positive for *B. duncani*, may actually be infected with *B. odocoilei* or another *Babesia* spp. Based on a combination of clinical manifestations and molecular confirmation, we provide causation of babesial infection and substantive evidence that *B. odocoilei* is one of several *Babesia* species worldwide that cause human babesiosis. Clinicians need to recognize that human babesiosis caused by *B. odocoilei* can be virulent and persistent in humans.

## Figures and Tables

**Table 1:** Dataset for human subjects infected with *Babesia odocoilei*, Ontario, Canada.

Subject	Age	Gender	Tick Bite	Lab Testing	Prior Treatment	Symptoms	GenBank Accession No.
1	23	female	not recalled	none, clinical diagnosis	atovaquone, symptoms unresolved	-night sweats -chills -fevers -profound fatigue -increased thirst -muscle aches -sleep disturbance	MW368483 -near match to type strains of *B. odocoilei*
2	74	male	*I. scapularis*, nymph	Yes, positive for *Babesia duncani* by serology; positive for *Babesia* by F.I.S.H.	atovaquone- azithromycin, symptoms unresolved; atovaquone-proguanil, symptoms unresolved	-night sweats -chills -fevers -profound fatigue -increased thirst -muscle aches -sleep disturbance	MW368482 -near match to type strains of *B. odocoilei*

## Data Availability

Data was provided by each subject by filling out a questionnaire and signing a consent form before blood was drawn at the medical clinic.
